# The application of the nnU-Net-based automatic segmentation model in assisting carotid artery stenosis and carotid atherosclerotic plaque evaluation

**DOI:** 10.3389/fphys.2022.1057800

**Published:** 2022-12-06

**Authors:** Ying Zhu, Liwei Chen, Wenjie Lu, Yongjun Gong, Ximing Wang

**Affiliations:** ^1^ First Clinical Medical College, Soochow University, Suzhou, China; ^2^ Department of Radiology, School of Medicine, Tongren Hospital, Shanghai Jiao Tong University, Shanghai, China; ^3^ Department of Radiology, The First Affiliated Hospital of Soochow University, Suzhou, China

**Keywords:** nnU-Net, automatic segmentation, computed tomography angiography, carotid artery stenosis, atherosclerotic plaque

## Abstract

**Objective:** No new U-net (nnU-Net) is a newly-developed deep learning neural network, whose advantages in medical image segmentation have been noticed recently. This study aimed to investigate the value of the nnU-Net-based model for computed tomography angiography (CTA) imaging in assisting the evaluation of carotid artery stenosis (CAS) and atherosclerotic plaque.

**Methods:** This study retrospectively enrolled 93 CAS-suspected patients who underwent head and neck CTA examination, then randomly divided them into the training set (N = 70) and the validation set (N = 23) in a 3:1 ratio. The radiologist-marked images in the training set were used for the development of the nnU-Net model, which was subsequently tested in the validation set.

**Results:** In the training set, the nnU-Net had already displayed a good performance for CAS diagnosis and atherosclerotic plaque segmentation. Then, its utility was further confirmed in the validation set: the Dice similarity coefficient value of the nnU-Net model in segmenting background, blood vessels, calcification plaques, and dark spots reached 0.975, 0.974 0.795, and 0.498, accordingly. Besides, the nnU-Net model displayed a good consistency with physicians in assessing CAS (Kappa = 0.893), stenosis degree (Kappa = 0.930), the number of calcification plaque (Kappa = 0.922), non-calcification (Kappa = 0.768) and mixed plaque (Kappa = 0.793), as well as the max thickness of calcification plaque (intraclass correlation coefficient = 0.972). Additionally, the evaluation time of the nnU-Net model was shortened compared with the physicians (27.3 ± 4.4 s vs. 296.8 ± 81.1 s, *p* < 0.001).

**Conclusion:** The automatic segmentation model based on nnU-Net shows good accuracy, reliability, and efficiency in assisting CTA to evaluate CAS and carotid atherosclerotic plaques.

## Introduction

Carotid artery stenosis (CAS), a common vascular disease, is one of the main pathological causes of ischemic stroke, which could be identified in nearly 20% of stroke patients; meanwhile, the prevalence of asymptomatic CAS ranges from 0.3% to 5.7% (Cheng et al., 2019; Carreira et al., 2020; Arasu et al., 2021; Heck and Jost, 2021). Concerning the initiation of CAS, the atherosclerotic plaque is nonignorable, which leads to narrowness or occlusion in the main neck arteries and further impacts the blood supply (Bos et al., 2021; Larson et al., 2021; Qaja et al., 2022). Due to the unique structure of the carotid artery (the Willis circle), a large number of patients are evaluated as asymptomatic CAS, who are easy to miss the best treatment timing and suffer from poor prognosis; hence, the accurate and timely diagnosis is essential (Carreira et al., 2020; US Preventive Services Task Force et al., 2021). At present, digital subtraction angiography (DSA) is considered to be the golden standard of CAS diagnosis; as it is an invasive surgery with a relatively high-dose contrast agent, many patients tend to choose computed tomography angiography (CTA), whose trauma and radiation quantity are milder (Dos Santos et al., 2016; Dohring et al., 2021). However, the CTA images still need clinicians to provide artificial judgment, which would take a relatively long time and the accuracy highly depends on the clinical experiences of physicians (Ghekiere et al., 2017). Therefore, some assistant diagnostic means [such as artificial intelligence (AI)] are generated to improve the efficiency and accuracy of CAS diagnosis, so as to promote the application of CTA in clinical practice.

U-net, a semantic segmentation algorithm of fully convolutional networks, is widely used in various imaging automatic segmentation and auxiliary diagnoses of many vascular diseases (Meshram et al., 2020; Zhou et al., 2021; Yin et al., 2022). For instance, a previous study develops a carotid segmentation model based on U-net to segment carotid bifurcation in CTA images (Zhou et al., 2021). No new U-net (nnU-Net), based on U-net technology, is a newly-developed deep learning neural network with a self-adapting function, which could handle various image properties and target structures (Isensee et al., 2021; Zhang et al., 2021b). Recently, the advantages of nnU-Net in medical image segmentation have been noticed in some studies (Heidenreich et al., 2021; Savjani, 2021). One study establishes a self-configuring nnU-Net model for automatic infarct segmentation in myocardial infarction patients; meanwhile, the infarct zone volumes obtained from this model and manual show good consistency (Heidenreich et al., 2021). Nevertheless, the application of nnU-Net in assisting CTA to evaluate CAS and to segment atherosclerotic plaque is rare.

Thus, this study established a nnU-Net-based automatic-segmentation model for CTA imaging in the training set and further verified its accuracy in the validation set, aiming to investigate its performance in assisting the evaluation of CAS and atherosclerotic plaque.

## Materials and methods

### Participants

This study retrospectively enrolled 93 patients with suspected CAS who underwent head and neck CTA examination between February 2021 and November 2021. The inclusion criteria were: 1) patients with suspected CAS, and had typical symptoms including transient monocular amaurosis or visual field defect, aphasia, limb numbness, clumsiness of motion; 2) had two or more risk factors for stroke, including hypertension, atrial fibrillation, diabetes, dyslipidemia, smoking, overweight, and history of transient ischemic attack; 3) underwent head and neck CTA examination; 4) had CAS and plaque confirmed by CTA. The exclusion criteria were: 1) history of interventional or surgical treatment, such as carotid stenting or carotid endarterectomy; 2) had cervical hemangioma or carotid artery vascular malformation; 3) CTA images were unclear or incomplete and insufficient for subsequent image processing; 4) missing clinical data. All 93 patients with suspected CAS were randomly divided into a training set (N = 70) and a validation set (N = 23) in a 3:1 ratio. The Institutional Review Board of The First Affiliated Hospital of Soochow University approved this study. The written informed consents were obtained from all participants.

### Clinical data collection

Clinical data of all patients were obtained through Electronic Health Record System, including age, gender, BMI (body mass index), smoke, hypertension, hyperlipidemia, diabetes, CKD (chronic kidney disease), CVD (cardiovascular disease), education level, marriage status, and place of residence.

### Computed tomography angiography image acquisition

The CTA examination was performed using a Toshiba 320-row-detector spiral computed tomography (CT) scanner (Aquilion ONE, Toshiba Medical Systems, Tokyo, Japan). The patients were placed in a supine position during the examination and were scanned at the end of the expiration when holding their breath. The parameters of scanning were as follows: reference 186 mAs, tube voltage 100 kV, pitch 0.8, rotation time 0.5 s, scanning time 3–4 s, delay time 2 s, collimator width 160 mm × 0.5 mm. Scan range was from the aortic arch to the level of both eyes, and the localization line was at the ascending aortic arch and the skull base, respectively. After completion of the conventional neck scan, the iodinated contrast media, iopamidol injection (Isovue-M^®^, United States) was used for the enhanced scan. A 30–45 ml bolus of non-ionic contrast material was injected by a dual barrel high-pressure injector at a flow rate of 4.5–5.0 ml/s with the threshold set at 180 HU in the descending aorta. Then, the scan raw data was transferred to the Vitrea (Vital Images, Minnetonka, MN, United States) software for post-processing. After the bone images were subtracted, the carotid vessels were reconstructed by image post-processing such as raw map, volumetric imaging, multi-planar reconstruction, and maximum density projection.

### Physicians’ annotations

The luminal stenosis location, plaque location, and plaque type (calcified plaque, non-calcified plaque, and mixed plaque) on CTA images of all patients were marked by two senior professional radiologists. Besides, the number of calcified plaques, non-calcified plaques, mixed plaques, the maximum calcified plaque thickness, and overall evaluation time was recorded. The plaques were segmented and labeled by the ITK-Snap tool (available at http://www.itksnap.org/pmwiki/pmwiki.php?n=Downloads.SNAP3). In case of disagreement, the two doctors discussed and decided on the best results.

### Carotid artery stenosis and its severity evaluation

The percentage of stenosis was calculated *via* the diameter measurements and the North American Symptomatic Carotid Endarterectomy Trial (NASCET) formula (North American Symptomatic Carotid Endarterectomy Trial Collaborators et al., 1991). Then the stenosis degree (if multiple stenoses were present, the most severe one was applied) was classified according to the Society of Cardiovascular Computed Tomography (SCCT) classification (American College of Cardiology Foundation et al., 2012). In detail, stenosis degree was classified as follows: 0%, no stenosis; 1%–49%: mild stenosis; 50%–69%: moderate stenosis; 70%–99%: severe stenosis; and 100%: blocking. Meanwhile, the occurrence of CAS was defined as “≥1% stenosis.”

### No new U-net model development

After enhancing and normalizing the images, the annotated images were thresholded for binarization. Combined with threshold segmentation and region growing algorithm, the common carotid artery to the extracranial segment of the internal carotid artery was segmented. The marked images in the training set were used for the development of a nnU-Net model, introducing residual convolution and null convolution modules. After forming the algorithm, the model was tested in the validation set. The principle of the nnU-Net deep neural network framework referred to the previous studies (Isensee et al., 2021). In the nnU-Net network, the size of the input and output 4D image block was 2 × 48 × 160 × 160. The encoding and decoding stages both contained five layers. In the coding phase, the first layer contained two 3 × 3 × 3 convolutions and two Leaky Linear Units (ReLU), and other layers contained a 3 × 3 × 3 convolution and a Leaky ReLU in turn. Except for the last convolution layer, each layer was followed by a 3 × 3 × 3 down sampling convolution with strides of 2 in each dimension. The channel number of the first convolution layer was set to 30, and the following convolution layer doubled the number of channels in turn. In the decoding phase, the five deconvolution layers with a size of 3 × 3 × 3 and stride size of 2 were deployed corresponding to the five down-sampling convolution layers. Every up-convolution layer was followed by two 3 × 3 × 3 convolutions. Each convolution layer except for the last layer halved the number of featuremaps channels in turn. The last layer reduced the number of output channels to the number of labels using a 1 × 1 × 1 convolution kernel. Five shortcut connections from layers of equal resolution in the analysis path transmitted the essential high-resolution features to the synthesis path. In the training step, the batch size was set to 4. Adam optimizer with an initial learning rate of 3e-4 was used to optimize network weight. The network was trained 21,900 times in 300 epochs. The detailed framework of the nnU-Net model was presented (Supplementary Figure S1). The coronal and axial imaging presentations of plaque segmentation assessed by the nnU-Net model and physicians in the training set (Supplementary Figures S2A–F) and the validation set (Supplementary Figures S3A–F) were displayed. The green area represented calcification plaques; the red area represented vascular lumens; the blue area represented dark spots.

The performance of the nnU-Net model in segmenting carotid plaque calcification was evaluated *via* area accuracy. The manually marked carotid plaque calcification areas in the dataset were used as the standard and compared with the results of the nnU-Net model automatic segmentation. Then the Dice similarity coefficient (DSC) was assessed as an evaluation index. DSC was a common measurement for assessing the similarity between manual and automatic segmentation; DSC ≥ 0.8 was considered a high similarity (Dionisio et al., 2021). The specific formula was as follows:

$$DSC=\frac{SEG\cap GT}{SEG+GT}\times 2$$

where *SEG* = segmented picture; *GT* = ground truth. In this study, SEG represented the calcified area obtained by nnU-Net model automatic segmentation and GT represented the calcified area obtained by manual segmentation.

### Statistical analysis

The statistical analyses were performed *via* SPSS v27.1 (IBM Corp., United States). The graphs were mapped by R V.4.0.5 (ggplot2 package, available at www.r-project.org) and GraphPad Prism v8.01 (GraphPad Software Inc., United States). The comparison of variables between the training set and validation set was evaluated through Student’s *t*-test, Chi-square test, Fisher exact test or Mann-Whitney *U* test. The consistency between the nnU-Net model and physicians in evaluating CAS was assessed through the Kappa coefficient test (Kappa ≥ 0.7 was considered highly consistent, 0.4 ≤ Kappa < 0.7 was considered moderately consistent, Kappa < 0.4 was considered weakly consistent). The consistency between the nnU-Net model and physicians in measured max thickness of calcification plaque was evaluated through the intraclass correlation coefficient (ICC) test. A *p* value < 0.05 indicated statistical significance.

## Results

### Clinical characteristics

**FIGURE 1:**
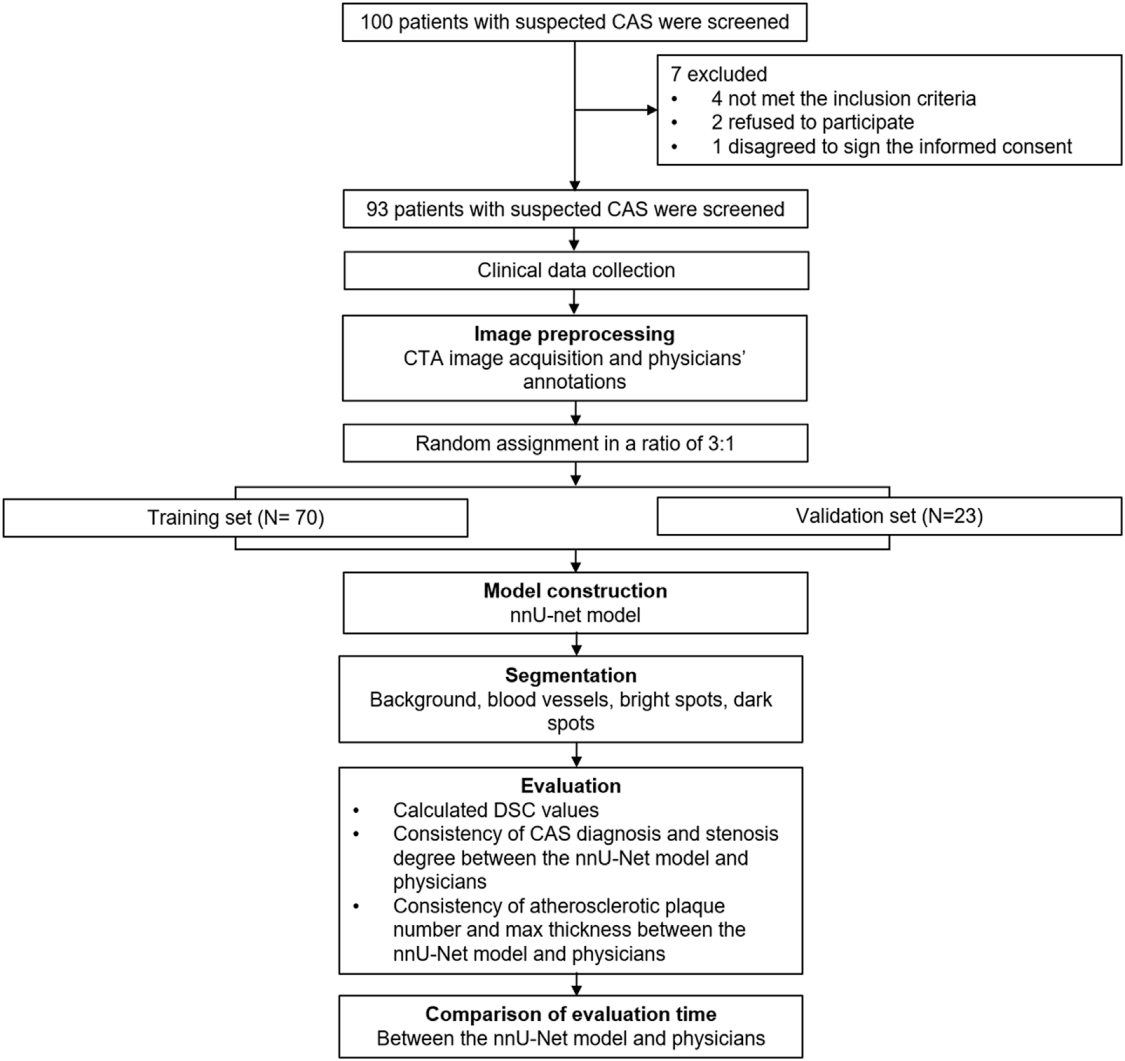
Study flow.

The whole process of this study was presented (Figure 1). A total of 93 patients with a mean age of 62.1 ± 8.3 years were randomly divided into the training set (N = 70) and validation set (N = 23) in a 3:1 ratio, whose mean age was 61.6 ± 8.5 years and 63.7 ± 7.8 years, respectively (Table 1). Concerning gender, the total patients consisted of 37 (39.8%) females and 56 (60.2%) males; besides, there were 29 (41.4%) females and 41 (58.6%) males in the training set, 8 (34.8%) females and 15 (65.2%) males in the validation set. Notably, all clinical characteristics were of no difference between the training set and validation set, including age, gender, BMI, smoke, hypertension, hyperlipidemia, diabetes, CKD, CVD, education, marriage status, and location (all *p* > 0.050). The detailed clinical characteristics were listed in Table 1.

**TABLE 1:** Clinical characteristics.

Items	Total patients (N = 93)	Training set (N = 70)	Validation set (N = 23)	*p* value*
Age (years), mean ± SD	62.1 ± 8.3	61.6 ± 8.5	63.7 ± 7.8	0.287
Gender, No. (%)				0.572
Female	37 (39.8)	29 (41.4)	8 (34.8)	
Male	56 (60.2)	41 (58.6)	15 (65.2)	
BMI (kg/m^2^), mean ± SD	23.9 ± 2.9	24.1 ± 3.0	23.4 ± 2.3	0.276
Smoke, No. (%)				0.245
No	35 (37.6)	24 (34.3)	11 (47.8)	
Yes	58 (62.4)	46 (65.7)	12 (52.2)	
Hypertension, No. (%)				0.559
No	24 (25.8)	17 (24.3)	7 (30.4)	
Yes	69 (74.2)	53 (75.7)	16 (69.6)	
Hyperlipidemia, No. (%)				0.234
No	62 (66.7)	49 (70.0)	13 (56.5)	
Yes	31 (33.3)	21 (30.0)	10 (43.5)	
Diabetes, No. (%)				1.000
No	74 (79.6)	56 (80.0)	18 (78.3)	
Yes	19 (20.4)	14 (20.0)	5 (21.7)	
CKD, No. (%)				0.726
No	82 (88.2)	61 (87.1)	21 (91.3)	
Yes	11 (11.8)	9 (12.9)	2 (8.7)	
CVD, No. (%)				0.400
No	67 (72.0)	52 (74.3)	15 (65.2)	
Yes	26 (28.0)	18 (25.7)	8 (34.8)	
Education, No. (%)				0.555
Primary school or below	23 (24.7)	16 (22.9)	7 (30.4)	
Junior high school	29 (31.2)	22 (31.4)	7 (30.4)	
High school	25 (26.9)	20 (28.6)	5 (21.7)	
University or above	16 (17.2)	12 (17.1)	4 (17.4)	
Marriage status, No. (%)				0.864
Married	66 (71.0)	50 (71.4)	16 (69.6)	
Divorced or widowed or single	27 (29.0)	20 (28.6)	7 (30.4)	
Location, No. (%)				1.000
Urban	12 (12.9)	9 (12.9)	3 (13.0)	
Rural	81 (87.1)	61 (87.1)	20 (87.0)	

SD, standard deviation; BMI, body mass index; CKD, chronic kidney disease; CVD, cardiovascular disease.

*Represented the *p* value for comparison of variables between the training set and validation set.

### The segmentation performance of the no new U-net model

In the training set, the nnU-Net model achieved the DSC value of 0.977, 0.962, 0.791, and 0.489 in segmenting background, blood vessels, calcification plaques, and dark spots. Meanwhile, the respective DSC value of the nnU-Net reached 0.975, 0.974, 0.795, and 0.498 in the validation set (Table 2).

**TABLE 2:** The performance of the nnU-Net model.

Total patients (N = 93)	Dice similarity coefficient			
	Background	Blood vessels	Calcification plaques	Dark spots
Training set (N = 70)	0.977	0.962	0.791	0.489
Validation set (N = 23)	0.975	0.974	0.795	0.498

### Consistency of carotid artery stenosis diagnosis and stenosis degree evaluation between the no new U-net model and physicians

The nnU-Net model and physicians displayed good consistency in assessing CAS both in the training set (Kappa = 0.860, *p* < 0.001) and validation set (Kappa = 0.893, *p* < 0.001) (Table 3). Also, the nnU-Net model exhibited a pleasing consistency with the physicians in evaluating stenosis degree both in the training set (Kappa = 0.874, *p* < 0.001) and the validation set (Kappa = 0.930, *p* < 0.001) (Table 4).

**TABLE 3:** Consistency between nnU-Net model and physicians in evaluating carotid artery stenosis in training and validation sets.

No. (%)	Assessed by nnU-Net model			Kappa coefficient	*p* value
		Non-stenosis	Stenosis		
Training set (N = 70)					
Assessed by physicians	Non-stenosis	48 (68.6)	1 (1.4)	0.860	<0.001
	Stenosis	3 (4.3)	18 (25.7)		
Validation set (N = 23)					
Assessed by physicians	Non-stenosis	16 (69.6)	1 (4.3)	0.893	<0.001
	Stenosis	0 (0.0)	6 (26.1)		

**TABLE 4:** Consistency between nnU-Net model and physicians in evaluating carotid artery stenosis degree in training and validation sets.

No. (%)	Assessed by nnU-Net model					Kappa coefficient	*p* value
		No stenosis	Mild stenosis	Moderate stenosis	Severe stenosis		
Training set (N = 70)							
Assessed by physicians	No stenosis	31 (44.3)	1 (1.4)	0 (0.0)	0 (0.0)	0.874	<0.001
	Mild stenosis	0 (0.0)	16 (22.9)	1 (1.4)	0 (0.0)		
	Moderate stenosis	0 (0.0)	3 (4.3)	12 (17.1)	0 (0.0)		
	Severe stenosis	0 (0.0)	0 (0.0)	1 (1.4)	5 (7.1)		
Validation set (N = 23)							
Assessed by physicians	No stenosis	13 (56.5)	0 (0.0)	0 (0.0)	0 (0.0)	0.930	<0.001
	Mild stenosis	0 (0.0)	3 (13.0)	1 (4.3)	0 (0.0)		
	Moderate stenosis	0 (0.0)	0 (0.0)	3 (13.0)	0 (0.0)		
	Severe stenosis	0 (0.0)	0 (0.0)	0 (0.0)	3 (13.0)		

### Consistency of atherosclerotic plaque number and max thickness evaluation between the no new U-net model and physicians

**FIGURE 2:**
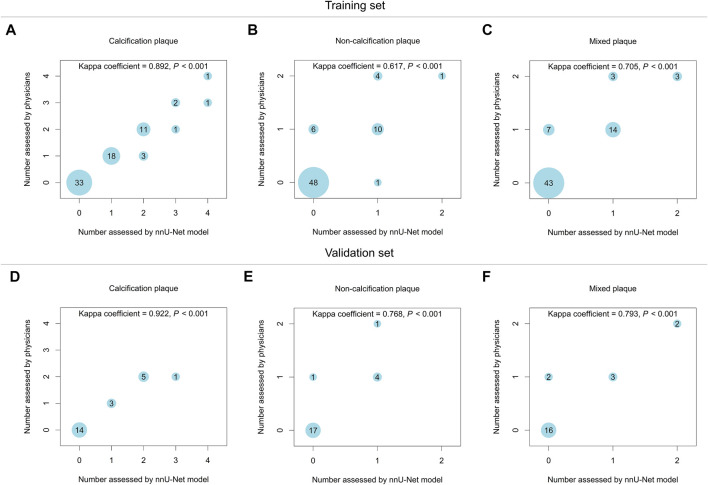
The nnU-Net model showed good consistency of atherosclerotic plaque number evaluation with physicians in the training set and validation set. The consistency between the nnU-Net model and physicians in evaluating the number of calcification plaque **(A)**, non-calcification plaque **(B)**, and mixed plaque **(C)** in the training set. The consistency between the nnU-Net model and physicians in evaluating the number of calcification plaque **(D)**, non-calcification plaque **(E)**, and mixed plaque **(F)** in the validation set.

In the training set, the nnU-Net model and physicians showed good consistency in evaluating the number of calcification plaque (Kappa = 0.892, *p* < 0.001) and the number of mixed plaque (Kappa = 0.705, *p* < 0.001), while they only displayed a moderate consistency in evaluating the number of non-calcification plaque (Kappa = 0.617, *p* < 0.001) (Figures 2A–C). Besides, in the validation set, the bubble plots showed good consistency between the nnU-Net model and physicians in evaluating the number of calcification plaque (Kappa = 0.922, *p* < 0.001), the number of non-calcification plaque (Kappa = 0.768, *p* < 0.001), and the number of mixed plaque (Kappa = 0.793, *p* < 0.001) (Figures 2D–F). The above data suggested that the nnU-Net model achieved the highest consistency with physicians in evaluating calcification plaque number, followed by mixed plaque number, and the lowest in non-calcification plaque number.

**FIGURE 3:**
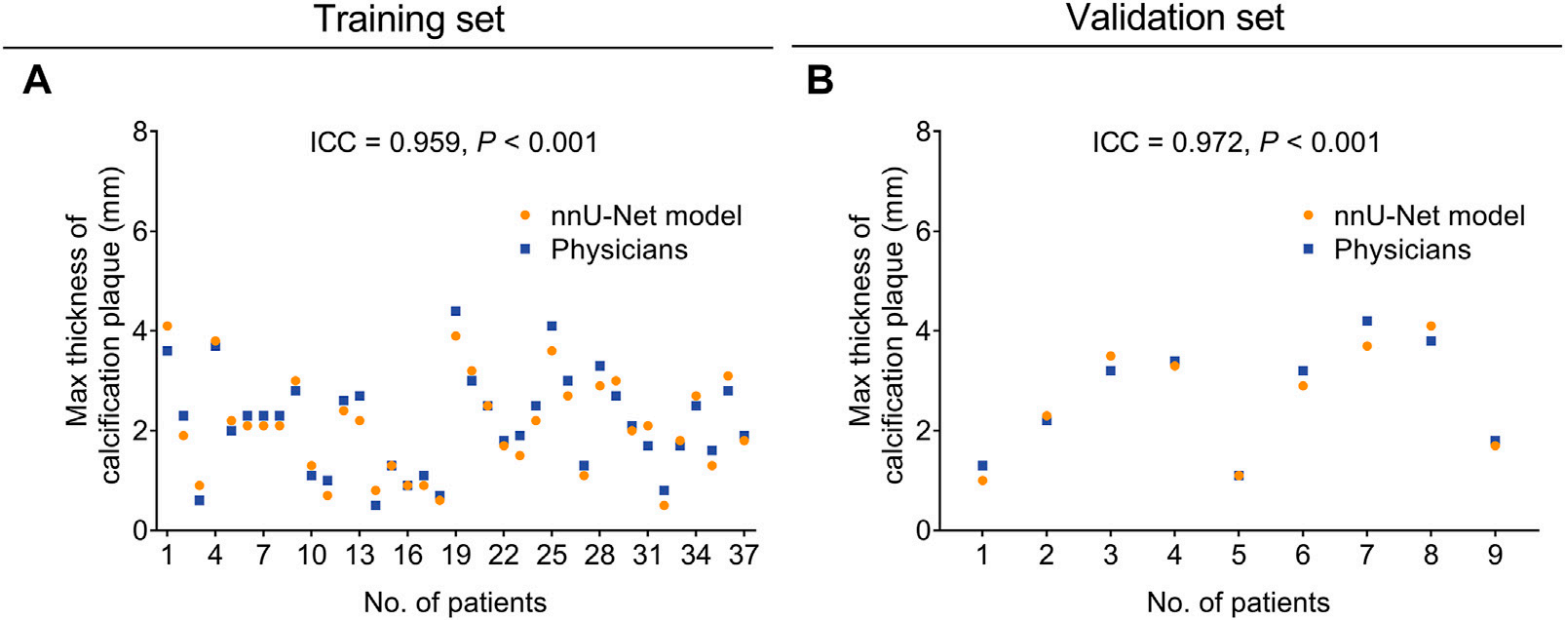
The nnU-Net model showed good consistency of calcification plaque max thickness evaluation with physicians in the training set and validation set. The consistency between the nnU-Net model and physicians in evaluating the max thickness of calcification plaque in the training set **(A)** and the validation set **(B)**.

Concerning the evaluation of calcification plaque max thickness, the nnU-Net model also achieved good consistency with physicians both in the training set (ICC = 0.959, *p* < 0.001, Figure 3A) and the validation set (ICC = 0.972, *p* < 0.001, Figure 3B).

### Comparison of evaluation time between the no new U-net model and physicians

**FIGURE 4:**
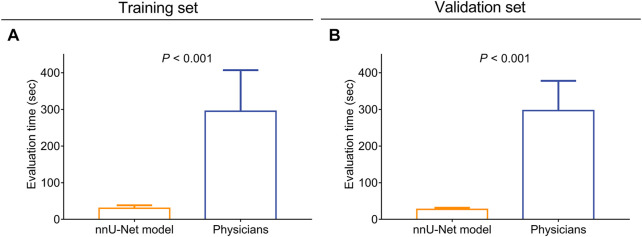
The nnU-Net model took less time for CAS and atherosclerotic plaque evaluation compared with physicians. Comparison of the evaluation time between the nnU-Net and physicians in the training set **(A)** and the validation set **(B)**.

The evaluation time of the nnU-Net model was shortened compared with the physicians both in the training set (30.2 ± 8.4 s vs. 295.0 ± 111.9 s, *p* < 0.001, Figure 4A) and the validation set (27.3 ± 4.4 s vs. 296.8 ± 81.1 s, *p* < 0.001, Figure 4B).

## Discussion

U-Net has been widely applied in medical imaging auxiliary diagnoses and vessel segmentation for several cardiovascular and cerebrovascular diseases since its development (Shelhamer et al., 2017; Dong et al., 2021; Zhao et al., 2021; Yin et al., 2022). For instance, a previous study develops a U-Net-based deep learning model to automatically segment coronary arteries in invasive coronary angiography, whose average DSC reaches 0.889, proving its potency in auxiliary coronary artery disease diagnosis (Zhao et al., 2021). However, as a newly-established algorithm based on three U-net architectures, the clinical application of the nnU-Net model in assisting CTA to diagnose CAS has not been studied yet. In this study, the nnU-Net model showed good performance in blood vessel segmentation with a DSC value of 0.977 in the training set and 0.975 in the validation set, which was numerically superior compared to the aforementioned study (Zhao et al., 2021). A probable reason might be that: U-Net had some inevitable shortcomings, including the inability to extract good features, insufficient high-resolution contour information, and the asymmetry between edge-cutting form and feature image (Li et al., 2022). While nnU-Net realized optimization of preprocessing, training, and data post-processing, which avoided ambiguity in contour segmentation (Zhang et al., 2021a). Therefore, the blood-vessel segmenting performance of the nnU-Net model was better than the U-Net model. Additionally, the consistency of CAS diagnosis and stenosis degree evaluation between the nnU-Net model and physicians was also satisfying, which might also contribute to its accurate blood-vessel segmenting performance. Besides, the data set was divided into the training set and validation set in a 3:1 ratio, which could be explained by that: the data set of this study was relatively small, and the majority of data should be applied for model establishment to reduce the risk of overfitting.

Apart from the blood vessels, the exact segmentation of atherosclerotic plaque is also essential for CAS diagnosis (Kondakov and Lelyuk, 2021; Kowara and Cudnoch-Jedrzejewska, 2021). However, the calcified area frequently exists shadow artifacts and “blooming artifacts,” which would interfere with the diagnosing accuracy of AI-assisted diagnosis under CTA and cause detection errors (Jinnouchi et al., 2020). While, in the present study, the DSC value of the nnU-Net model in segmenting calcification plaques reached 0.791 (in the training set) and 0.795 (in the validation set); meanwhile, the consistency between the nnU-Net model with physicians was also pleasing in evaluating atherosclerotic plaque numbers and the max thickness of calcification plaque. The results reflected the good atherosclerotic plaque-segmentation performance of the nnU-Net model, which could be explained as follows: the nnU-Net algorithm sharpened the edge of calcification components during the imaging processing, meanwhile, its deep learning methods on the edge features of calcification plaque and other components improved the segmentation accuracy (Li et al., 2021). Unfortunately, the DSC value of the nnU-Net model in segmenting dark spots was only 0.489 and 0.498 in the training set and the validation set, correspondingly, suggesting that the network structure needed further improvement.

Considering that the traditional manual image-marking is time-consuming and inefficient, which greatly occupies the diagnosis time of clinicians, the time-saving advantage of nnU-Net is highlighted in previous studies (Mihelic et al., 2021). For instance, a previous study reports that the nnU-Net model takes approximately 20 s for the whole breast segmentation and nearly 15 s for the fibroglandular tissue segmentation under the dynamic contrast-enhanced magnetic resonance images (Huo et al., 2021). Similarly, the current study also noticed that the evaluation time of the nnU-Net model was shortened compared with the physicians in both the training set (30.2 ± 8.4 s vs. 295.0 ± 111.9 s) and the validation set (27.3 ± 4.4 s vs. 296.8 ± 81.1 s). A possible explanation might be as follows: nnU-Net was an automated configuration, covering the entire segmentation without any manual decision, meanwhile, it contained simple execution rules which realized the simplified compute resources (Isensee et al., 2021; Tan et al., 2021). Consequently, owing to its flexible and reliable strategy, nnU-Net exhibited its convenience in CAS assessment and atherosclerotic plaque segmentation. Additionally, the evaluation time represented the mean value of 300 epochs, whose difference between the validation set and training set lacked statistical significance; hence, it could not be concluded that the feasibility and efficiency were improved after the training set.

Some limitations were noticed in this study. Firstly, the small sample size (N = 93) might lead to a relatively weak statistical power. Meanwhile, the small number of patients in the validation set would hinder the presentation of the efficacy of the nnU-Net-based automatic segmentation model. Thus, further studies with a larger data set were necessary. Secondly, this was a retrospective study whose selective bias was hard to avoid. Thirdly, in order to validate the universality of this model, external validation was necessary to conduct in further studies. Fourthly, this study enrolled CAS-suspected patients (but not CAS-confirmed or stroke patients) to generally investigate its potential in the cardio/cerebrovascular field; hence, further studies conducted in some specific diseases were warranted.

Collectively, the automatic segmentation model based on nnU-Net shows good accuracy, reliability, and efficiency in assisting CTA to evaluate CAS and carotid atherosclerotic plaques, implying the prospect of deep-learning AI technology in auxiliary imaging diagnosis.

## Data Availability

The original contributions presented in the study are included in the article/Supplementary Material, further inquiries can be directed to the corresponding authors.
