# Supplementary material for: The application of the nnU-Net-based automatic segmentation model in assisting carotid artery stenosis and carotid atherosclerotic plaque evaluation
Source: Front Physiol. 2022 Dec 6;13:1057800. doi: 10.3389/fphys.2022.1057800 (PMC9763590; doi:10.3389/fphys.2022.1057800)
Supplement: Supplementary file 1 [file DataSheet1.docx]

***Supplementary Materials***

***
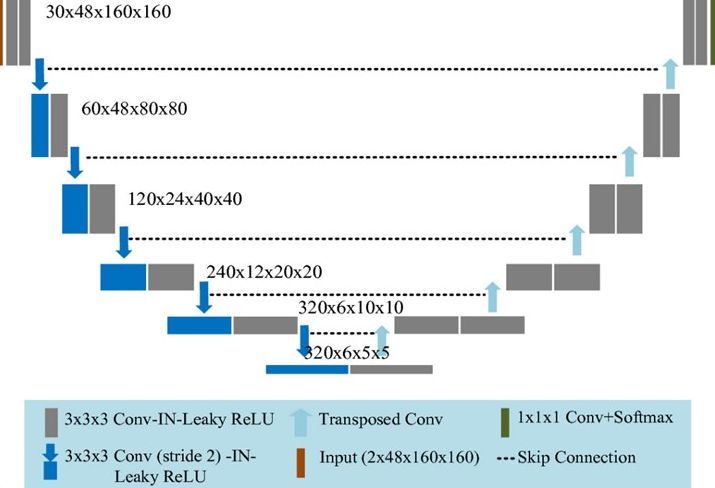
***

**Supplementary Figure 1.** The framework of the nnU-Net model.


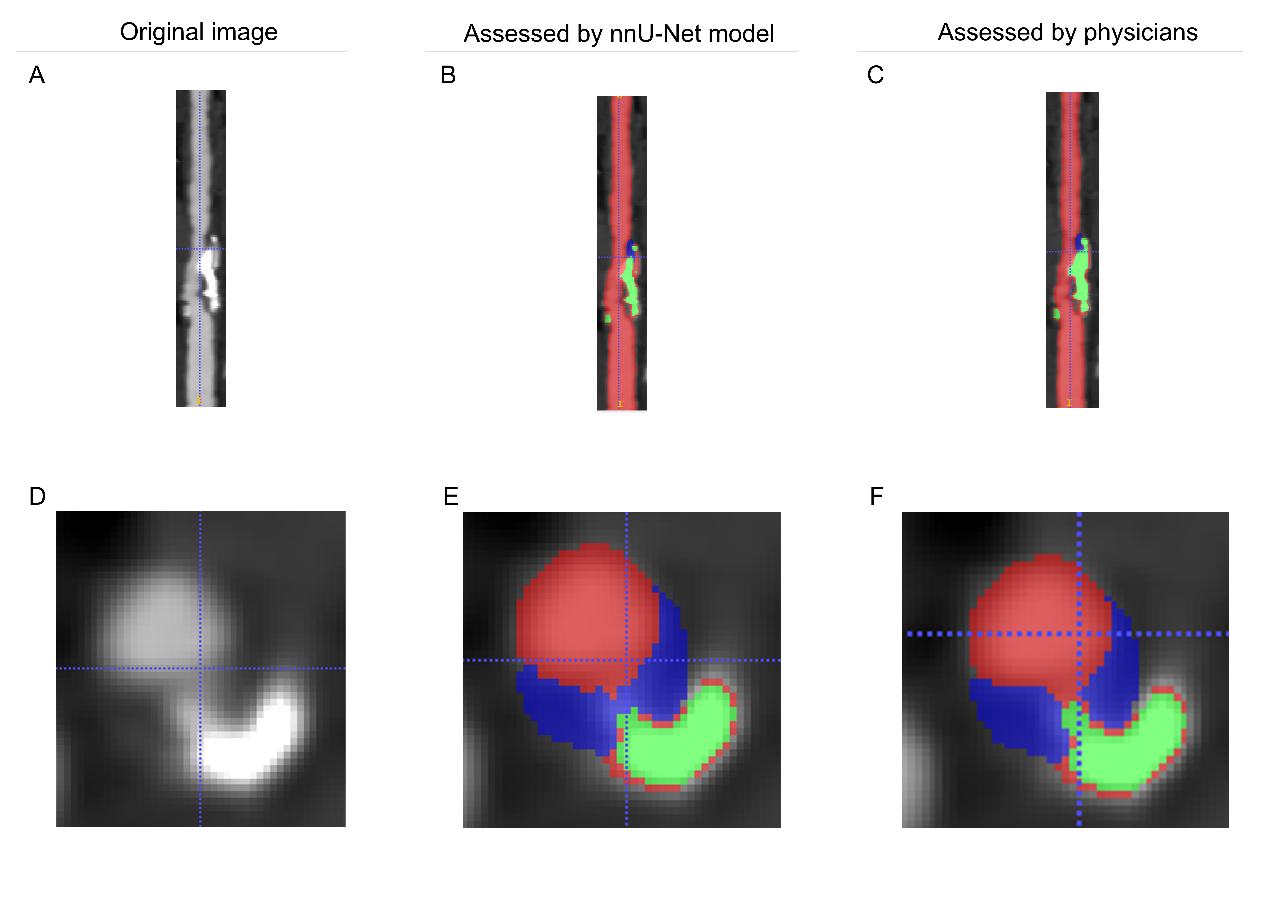


**Supplementary Figure 2.** The representative case of segmenting results in the training set. The coronal original image (**A**), the coronal image assessed by the nnU-Net model (**B**), the coronal image assessed by physicians (**C**), the axial original image (**D**), the axial image assessed by the nnU-Net model (**E**), and the axial image assessed by physicians (**F**) in the training set were exhibited.


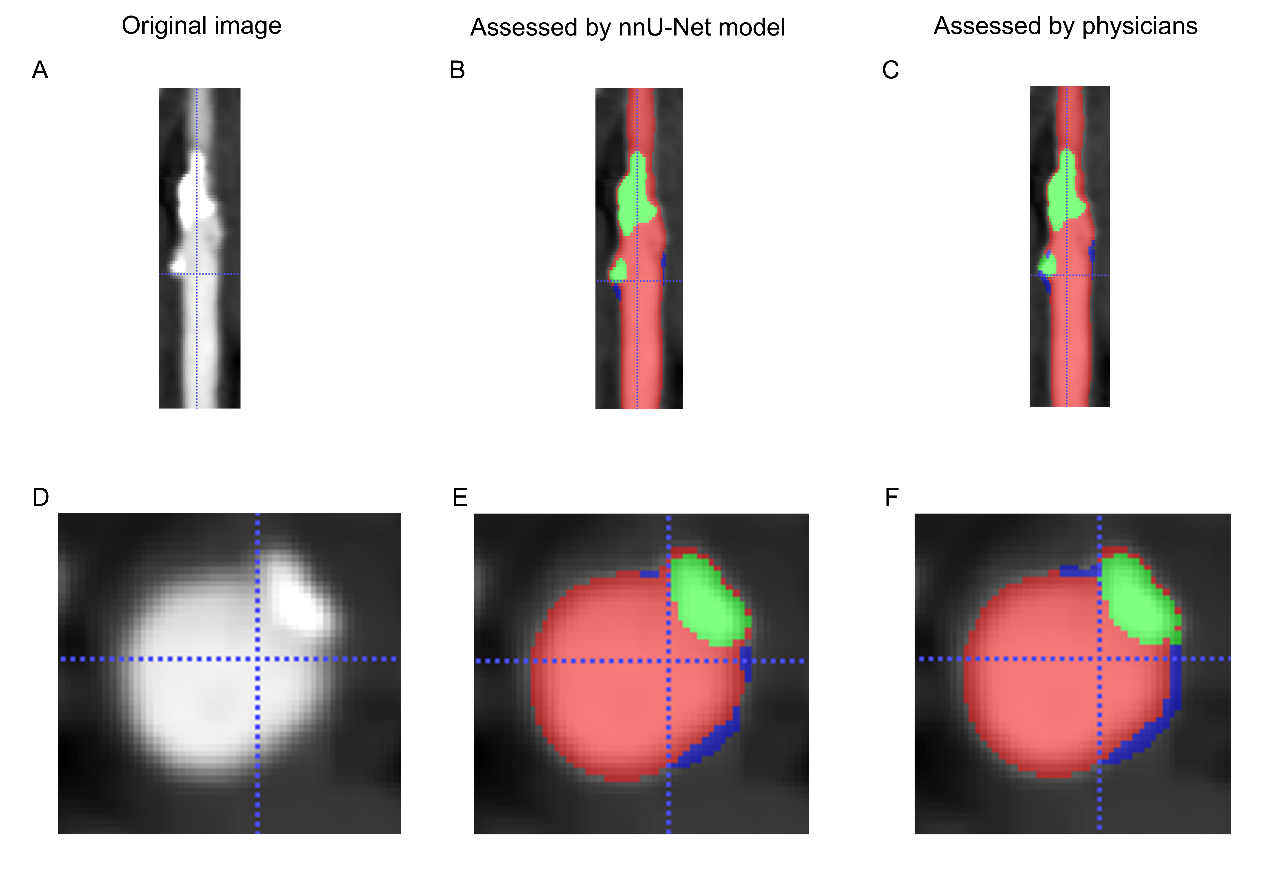


**Supplementary Figure 3.** The representative case of segmenting results in the validation set. The coronal original image (**A**), the coronal image assessed by the nnU-Net model (**B**), the coronal image assessed by physicians (**C**), the axial original image (**D**), the axial image assessed by the nnU-Net model (**E**), and the axial image assessed by physicians (**F**) in the validation set were exhibited.
